# Computerized chest radiograph analysis of air distribution after surfactant treatment of respiratory distress syndrome in extremely preterm infants

**DOI:** 10.1007/s00431-025-06466-1

**Published:** 2025-10-14

**Authors:** Julia Fall, Richard Sindelar, Malin Helenius, Eva Penno, Leif D. Nelin, Laszlo Markasz

**Affiliations:** 1 Department of Women’s and Children’s Health, Uppsala University Children’s Hospital, Uppsala University, 751 85 Uppsala, Sweden; 2 Department of Surgical Sciences, Radiology, Uppsala University, Uppsala, Sweden; 3 Nationwide Children’s Hospital, Ohio State University College of Medicine, Columbus, OH USA

**Keywords:** Surfactant, Extremely premature, Radiography, Chest X-ray, Outcomes

## Abstract

The early air distribution after surfactant instillation can have implications for the development of lung injury in extremely preterm newborns with respiratory distress syndrome (RDS). The objective of this study is to determine lung air distribution and focal heterogeneity after surfactant treatment of RDS, using computerized image analysis of chest radiographs (CXRs). This is a retrospective observational non-paired study where CXRs were assessed within 6 h after surfactant instillation in the surfactant-treated group [STG] (*n*  =  52; median 24^+4^; range 22^+0^–27^+5^ weeks gestation) and at the same postnatal age in the pre-surfactant group [PSG] (*n* = 8; 25^+3^; 23^+6^–26^+4^). Digital image analysis was performed by ImageJ of lung intercostal segments 2–7 bilaterally: mean pixel intensity (MPI) determines focal lung density and correlates negatively with lung aeration; focal heterogeneity in pixel intensity (FHPI) defines focal differences in aeration and correlates positively with overexpansion/atelectasis. MPI was higher in the left lung in STG than in PSG (*p* = 0.008). MPI was higher in the apical compared to the basal regions bilaterally, in both STG (right, *p* = 0.024; left, *p* = 0.015) and PSG (right, *p* = 0.047; left, *p* < 0.001). FHPI was higher on the right compared to the left 7th intercostal segment in STG (*p* = 0.006). FHPI of the right 7th segment was higher in STG than in PSG (*p* < 0.001).

*Conclusions*: In this retrospective observational study of CXRs in extremely preterm infants, the right lungs were more aerated than the left lungs after surfactant, whereas the basal regions were more aerated than the apical regions regardless of surfactant treatment. We hypothesize that increased focal heterogeneity in these more aerated regions after surfactant may reflect overexpansion/atelectasis, which may be secondary to an inhomogeneous surfactant distribution, an observation that needs to be further validated in future studies.

**Table:** 

**What is Known:** •*Surfactant treatment has a major impact on survival in extremely preterm infants (EPIs) with respiratory distress syndrome (RDS), but less is known about the achieved early air distribution and its possible implications for future lung outcomes.* **What is New:** •*Computerized image analysis of plain chest radiographs (CXRs) provides more detailed information on the air distribution than clinical radiological grading whereby the optimal time point and administration techniques for surfactant instillation can be evaluated in EPIs with RDS.*

**Supplementary Information:**

The online version contains supplementary material available at 10.1007/s00431-025-06466-1.

## Introduction

Despite advances in neonatal care, respiratory distress syndrome (RDS) remains a common cause of respiratory failure in preterm infants [1] and increases in incidence with decreasing gestational age (GA) [2]. RDS is caused by surfactant deficiency [3, 4] which leads to stiff lungs, atelectasis, ventilation-to-perfusion (V/Q) mismatch, hypoxia, and/or hypercapnia [2]. The uneven air distribution may also lead to overdistention of well-ventilated alveoli, which could potentially injure the lung epithelium [2]. The main radiographic finding in patients with RDS is poorly inflated lungs [5, 6]. Exogenous surfactant is now a well-established treatment for RDS, and diagnosis of RDS includes clinical assessment of work of breathing (WOB), inspired oxygen requirement, and lung volumes on chest radiograph (CXR) [7]. Previously, prophylactic surfactant treatment was recommended for extremely preterm infants, but with improvements in administering nasal constant positive airway pressure (nCPAP), a more restrictive initial surfactant strategy has been proposed, with recommended early rescue surfactant if the lung condition continues to deteriorate [7]. With less invasive strategies for surfactant administration, a more liberal use of surfactant treatment in extremely preterm infants has been established, which warrants further evaluation [8].

Respiratory distress syndrome is usually responsive to initial treatment with surfactant and CPAP, but many of these preterm infants still go on to develop bronchopulmonary dysplasia (BPD), the chronic lung disease of prematurity that is associated with both increased mortality and morbidity [9]. Some of the risk factors associated with the development of BPD are low GA, the need for, and the duration of, invasive mechanical ventilation (MV), chorioamnionitis, and fetal growth restriction [9]. Even though a recent Swedish National study of preterms born between 22 and 26 weeks’ GA observed increased survival and a decrease in severe BPD (sBPD) over time (2004–2007 vs 2014–2016), in the most recent epoch, 68% of these infants still had some degree of BPD, and 23% developed sBPD [10].

In 1991, Goldsmith et al. investigated the immediate improvement in lung volume after surfactant instillation by studying lung mechanics in preterm infants [11]. They proposed that the immediate improvement in lung volume after surfactant was due to increased distention of already open alveoli rather than alveolar recruitment and hypothesized that surfactant may contribute to barotrauma and lung injury [11]. Since this report, few studies have looked at the air distribution in the lungs after surfactant administration, and most of these studies have been performed in animal models [12–14].

In view of a possibly surfactant-induced difference in aeration of the lungs, a more detailed assessment of CXRs early after surfactant instillation may define early signs of lung heterogeneity and possibly have implications for the development of lung injury. We hypothesize that surfactant is potentially unevenly distributed in the lung, leading to a combination of over-expansion and atelectasis. The aim of our study was to investigate the lung aeration after surfactant treatment of RDS in the lungs of extremely preterm infants, using computerized image analysis of CXRs and correlating aeration with clinical parameters.

## Materials and methods

### Patients

This observational study included extremely low gestational age newborns (ELGANs; GA 22^+0^–27^+6^ weeks) born at the Neonatal Intensive Care Unit (NICU) at Uppsala University Children’s Hospital in Uppsala, Sweden, between January 1, 2021, and December 31, 2022, who were treated with surfactant (Curosurf®; Chiesi; Parma, Italy). The study was approved by the Swedish Ethical Board in Uppsala, DNR 2023–03297-01.

Of the 66 infants meeting criteria, 28 were female (42%) and 38 male (58%). All pregnancies were evaluated by ultrasound examination at gestational weeks 14–17. The indication for surfactant treatment was bradycardia directly after birth or clinical signs of RDS, such as rising oxygen requirement with fraction of inspired oxygen (FiO_2_) > 30% and increased WOB. Infants with GA 22–24 weeks were treated prophylactically with surfactant immediately after birth. Surfactant was administered using the less invasive INtubation-SURfactant-Extubation technique [15, 16].

One infant was excluded because of early death (no record of CXR), and another was excluded due to requiring intubation for surgery and not for respiratory failure or surfactant treatment (Supplement 1). Any CXR prior to surfactant treatment and the CXR closest in time following, and not more than 6 h after, the first surfactant administration was used in this analysis. Of the 64 included infants, 8 had CXRs taken prior to surfactant instillation with no CXR taken within 6 h of surfactant treatment, and were defined as the pre-surfactant group (PSG). For the remaining 56 infants, 4 were excluded as their CXRs were taken more than 6 h after surfactant administration. The remaining 52 infants were the surfactant-treated group (STG) (Supplement 1). To ensure that all items of an observational study were reported, the STROBE checklist was used [17].

### Data collection

Patient data were collected from the medical records and included three sources: 1. the digital images of the chest radiographs (including time of imaging) were obtained from the radiological database Telerad; 2. clinical ventilatory data were collected from the patient data monitoring system Metavision®; and 3. additional patient data were collected from the national Swedish Neonatal Quality register (SNQ). Data were collected on day of life (DoL) 1 (first 24 h in life), 3, and 7, at three time points each day (08.00, 16.00, and 24.00). A digital image file of the frontal CXR was collected and saved for each patient. All CXR data were extracted and anonymized with a code. The parameters describing perinatal data were collected from SNQ, and the intensive care monitoring system (Metavision®) was used to extract ventilatory data.

ImageJ® (National Institute of Health, Bethesda, MD, USA) was used for digital image processing and analysis of CXRs. In total, 60 CXR images were analyzed. Since the CXRs were taken with different exposure times, resulting in different background intensities, the images were normalized to their pixel intensities. The pixel with maximum intensity level was selected from the site of the liver for each infant as a reference, and the intensity levels of all pixels were normalized to this reference point for each individual image. This normalization resulted in the reference point of the liver becoming the same intensity level in all infants, and the pixel intensities became comparable between images (Supplement 2**)**. A scale bar of 10 mm helped to normalize the image size for each image and make the area measurements between infants comparable. The multiplication with an individual size factor for each corresponding image resulted in the scale bars appearing with the same length in number of pixels (10 mm = 50 pixels) in all images after this adjustment (Supplement 2). All images (*n* = 60) were organized into the same stack (image size 2075 × 1170 pixels). Twelve regions of interest (ROIs), according to intercostal space 2 to 7 on the left (sin 2–7) and right (dex 2–7) lung, respectively, were selected manually for each infant (*n* = 720) by avoiding other structures than lung tissue (Supplement 2). The ROI Manager Tool was used to measure two parameters that described focal lung structure in the selected ROIs:

- **The mean pixel intensity (MPI)** of the corresponding ROI selection described the focal lung density: A higher MPI corresponded to a greater lung tissue density (lower index of aeration) in the lung segment (Supplement 3). MPI was calculated by ImageJ automatically, from the sum of the pixel intensity values of all the pixels in the ROI divided by the number of pixels.
- **The focal heterogeneity in pixel intensity (FHPI)** per ROI described the focal pattern of heterogeneity in density in the corresponding lung segment. Higher FHPI reflected a higher heterogeneity of aeration in the individual lung segment (Supplement 4), and the higher FHPI, the more uneven the distribution of air in that ROI. ImageJ generated FHPI from the standard deviation of the pixel intensity values used to generate the MPI.

To describe the heterogeneity in MPI and FHPI among the focal lung segments, the standard deviations of both MPI and FHPI values (STDV-MPI and STDV-FHPI) were calculated in Microsoft Excel. Higher STDV-MPI corresponded to higher differences in air content among the segments (Supplement 5). Higher STDV-FHPI corresponded to higher differences in the focal pattern of heterogeneities within the segments (Supplement 6). By interpreting all four parameters for the lung segments simultaneously, it was possible to predict precisely how the corresponding lungs appear in the chest radiogram. The measurement of the 7th intercostal segment in the right lung was excluded in 7 surfactant-treated infants due to overlapping of the liver.

###  Statistical analysis

The calculations and the statistics were performed in Microsoft® Excel version 16.78.3 and JASP version 0.95 (freeware, Univeristy of Amsterdam, Netherlands). Categorical infant characteristics and ventilatory long-term outcomes are presented as numbers (*n*) and percentages (%), and continuous infant characteristics are presented as median and interquartile range. Continuous ventilatory and long-term outcome data are presented as mean and standard deviation (SD). Student’s two-sided, heteroscedastic *t*-test was used on numeric variables, and Fisher’s exact test was used on dichotomous variables. Pearson’s regression analysis was performed to find correlations. A *p*-value of < 0.05 was considered significant. In case of multiple comparisons, the Bonferroni or Conover’s post hoc test was used to estimate the value of significance after repeated measures ANOVA for parametric values or Friedman test for non-parametric values, respectively.

## Results

Main infant characteristics are presented in Table 1. All included infants received surfactant treatment. The median number of administered doses in the STG was 2 (IQR 1–2 doses), whereas all infants in the PSG were given only one single dose (*p* < 0.001). The median time from surfactant administration to CXR was 106 min for the STG (IQR 94–132 min). No significant difference was observed in the median age at CXR between the STG and the PSG.

**Table 1:** Perinatal characteristics

	**Surfactant-treated group** ***n* = 52**	**Pre-surfactant group** ***n* = 8**	***p*-value**
	***n* (%)**	***n* (%)**	
Twins	8 (15)	0 (0)	0.004
Chorioamnionitis	0 (0)	0 (0)	
Preeclampsia/eclampsia	6 (12)	0 (0)	0.013
Prenatal steroids			
> 24 h before birth	20 (38)	4 (50)	0.841
< 24 h before birth	15 (29)	0 (0)	< 0.001
Male sex	30 (58)	4 (50)	0.711
PPROM 0–24 h	23 (44)	5 (63)	0.342
PPROM > 24 h–1 week	12 (23)	2 (25)	0.972
PPROM > 1 weeks	4 (12)	0 (0)	0.493
Cesarean section	26 (50)	5 (63)	0.606
	Median (Q1-Q3)	Median (Q1-Q3)	
Gestational age, weeks^+days^	24^+4^ (22^+1^–26^+4^)	25^+3^ (25^+1^–26^+4^)	0.241
Birthweight, gram	640 (498–915)	718 (656–796)	0.373
Apgar 5 min	6 (4–8)	9 (8–9)	< 0.001
	Median (Q1–Q3)	Median (Q1–Q3)	
Total number of surfactant doses	2 (1–2)	1 (1–1)	< 0.001
Age at first surfactant, minutes	6 (4–10)	587 (223–1726)	0.030
Age at CXR, minutes	118 (105–145)	108 (95–143)	0.194
Time from surfactant to CXR, minutes	106 (94–132)	0*	0.029

^*^All infants in this group received surfactant after the first CXR at a median time interval of 425 min (IQR 116–1033)

*PPROM* preterm premature rupture of the membranes, *CXR* chest X-ray

**Fig. 1:**
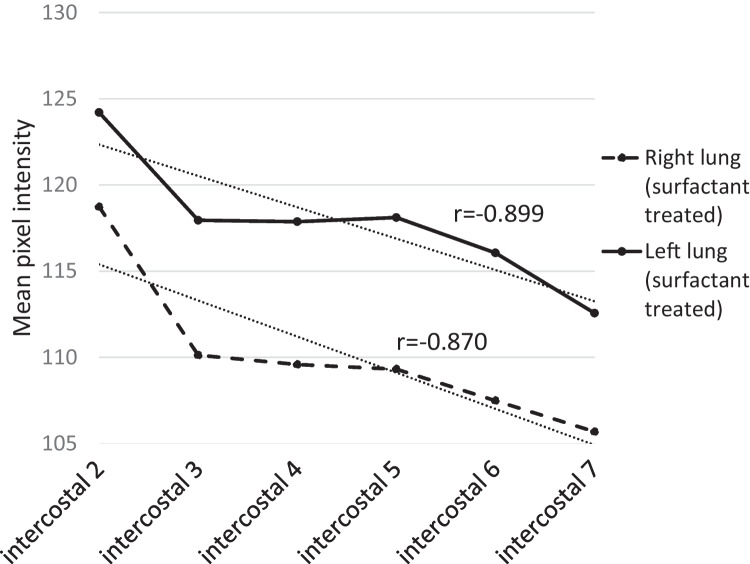
Mean values of mean pixel intensity (MPI) of each segment in surfactant-treated group. Significant linear correlation between intercostal space 2 and 7 was noted bilaterally (right lung, *r* =  − 0.870, *p* = 0.024; left lung, *r* =  − 0.899, *p* = 0.015). The left lung had higher MPI than the right (*p* = 0.008). There was no difference in the slopes of the lines between right and left lungs (right lung, *y* =  − 1,99x + 117,79; left lung, y =  − 1,75x + 123,71). There was a difference between intercostal space 2 and 7 (right and left side, *p* < 0.001). The measurement of the 7th intercostal segment was excluded in 7 infants (all from STG) due to poorly inflated lungs resulting in interference with the liver

The left lung in the STG had higher MPI than the right (*p* = 0.008) (Fig. 1). The mean MPI was also significantly higher in the apical lung regions compared to the basal lung regions, decreasing linearly (right, *r* = −0.870, *p* = 0.024; left, *r* = −0.899, *p* = 0.015). There was a significant difference in MPI on both the right and left sides between intercostal space 2 and 7 (*p* < 0.001) (Fig. 1).

**Fig. 2:**
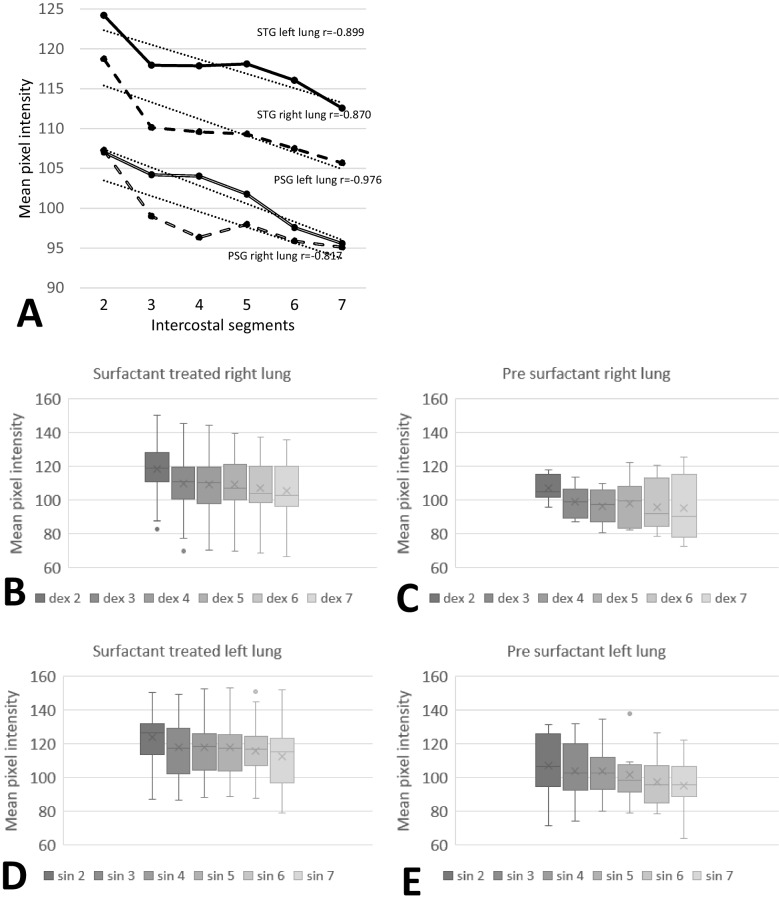
**A–E** MPI in surfactant-treated and pre-surfactant group. **A** The mean MPI values in the surfactant treated and pre-surfactant group. Linear correlation in the surfactant-treated group (right lung, r =  − 0.870, p = 0.024; left lung, r =  − 0.899, p = 0.014) and the pre-surfactant group (right lung, r =  − 0.817, p = 0.047; left lung, r =  − 0.976, p < 0.001). **B**, **C** Distribution of MPI (mean (X), lower and upper quartile, and minimum and maximum data value, outliers) in right lung segments of surfactant-treated and pre-surfactant groups, respectively. **D**, **E** Distribution of MPI (mean (X), lower and upper quartile, and minimum and maximum data value, outliers) in left lung segments of surfactant treated and pre-surfactant groups, respectively

The PSG showed the same correlation as the STG with a higher MPI in the apical regions compared to the basal regions, which decreased linearly (right lung, *r* = −0.817, *p* = 0.047; left lung, *r* = −0.976, *p* < 0.001; Fig. 2A). There was no difference in the mean MPI between the right and left lung in PSG (Fig. 2A). The mean MPI of the right and left lung was also higher in the STG (right lung, 110, SD 16; left lung, 118, SD 16) compared to the PSG (right lung, 98.6, SD 13; left lung, 102, SD 17) (*p* = 0.031 and *p* = 0.029, respectively) (Fig. 2A). The dispersion of MPI in the right and left lung segments was, however, generally larger for many of the lung segments in the PSG compared to the STG (Fig. 2B-E), especially the right 7th lung segment (Fig. 2B-C). No significant differences in STDV-MPI of the left and right lung fields were found within or between the surfactant-treated and PSG.

**Fig. 3:**
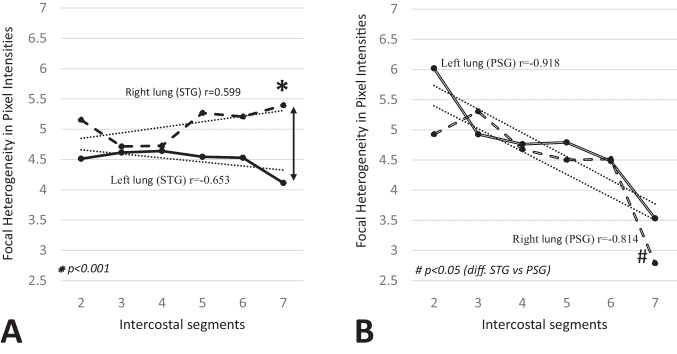
**A**, **B** The mean focal heterogeneity of pixel intensities (FHPI) in surfactant treated (STG) and pre-surfactant group (PSG). **A** The measurement of the 7th intercostal segment was excluded from seven infants due to poorly inhaled image resulting in interference with the liver. Linear regression was not significant for right or left side in STG. *Significant difference in FHPI between right and left 7th intercostal segment in STG (p = 0.006). **B** Significant linear correlation between intercostal segment 2 to 7 bilaterally in PSG (right lung, p < 0.05; left lung, p < 0.01).#Significant difference in FHPI of right 7th intercostal segment between the STG and PSG (p < 0.001)

The right 7th intercostal segment had significantly higher FHPI than the left 7th segment in the STG (*p* = 0.006) (Fig. 3A). FHPI in the right 7th segment of the STG was also significantly higher than in the PSG (*p* < 0.001) (Fig. 3B). In PSG, FHPI was higher in the apical regions, decreasing linearly towards the basal regions bilaterally (right lung, *r* = −0.814, *p* < 0.05; left lung, *r* = −0.918, *p* < 0.01) (Fig. 3B). No significant differences in STDV-FHPI of left and right lung fields were found within or between the STG and PSG.

**Fig. 4:**
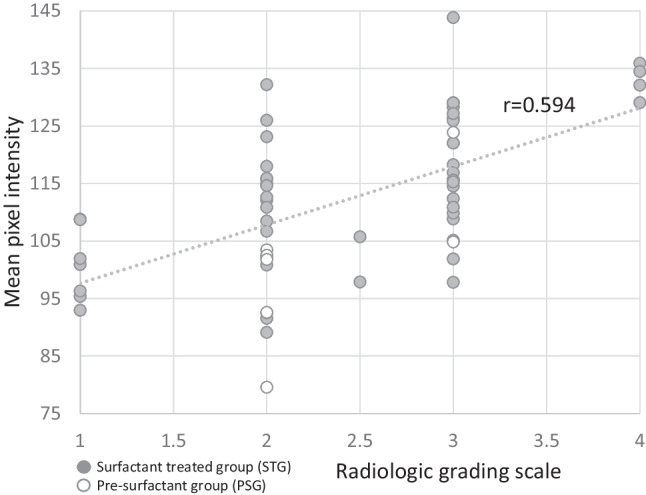
Correlation between MPI and radiological image grading scale. Orange points represent infants from the surfactant-treated group (n = 52). White points with orange circle represent infants from the pre-surfactant group (n = 8). A significant positive linear correlation (r = 0.594; p < 0.001) between radiological image grading and MPI was found (r = 0.594)

The computerized image analysis of all images (*n* = 60) was compared with an independent visual grading of RDS performed by two pediatric radiologists at the Radiology Department at Uppsala University Hospital. The radiologists performed an evaluation of the CXRs in general, without details on focal aeration, by using a scale from 1 to 4, where 4 was the most severe RDS. The grading by the radiologists was positively correlated with the computerized measurements of MPI performed in ImageJ (*r* = 0.594; *p* < 0.001) (Fig. 4). No correlation was found between their grading and FHPI.

For the infants in the STG, the number of infants on MV decreased during the first week and was lowest on day 7 compared to day 1 (*p* < 0.001) (Supplement 7 and 8). The tidal volume (VT) decreased between day 1 and 3 (*p* < 0.001) and increased between day 1 and 7 (*p* < 0.001), but did not differ significantly between day 3 and 7 (*p* = 1.000) (Supplement 8).

There was a significant positive linear correlation between MPI and mean airway pressure (MAP) on day 1 (*r* = 0.479; *p* < 0.001), where a higher MPI corresponded to a higher MAP on day 1 (Supplement 9). There was no significant correlation found for any of the other clinical parameters, including MAP on day 3 or 7, and FiO_2_, respiratory severity score, GA, BW, or VT on day 1, 3, or 7, with either MPI or FHPI (data not shown).

The mean MV duration was 17±31 days and mean length of stay was 75±51 days. Out of the 52 infants in the STG, 20 (38%) were diagnosed with BPD, of which 12 (23%) were diagnosed with sBPD. The mortality in the STG group was 17 (33%) (Supplement 10). Correlations between the different digital image analyses of the CXRs and BPD were not possible to evaluate due to the low number of infants.

## Discussion

In summary, we found that the mean pixel intensity (MPI) was lower in the right lung compared to the left lung, indicating a higher index of aeration in the right lung after surfactant. The apical regions of the lungs had a higher MPI, indicating a higher index of aeration in the basal regions of the lungs regardless of surfactant. Finally, focal heterogeneity in pixel intensity (FHPI) was higher in these more aerated regions after surfactant, indicating a higher heterogeneity of aeration, which could reflect overexpansion/atelectasis, which may be secondary to an inhomogeneous surfactant distribution.

The introduction of surfactant has had substantial importance for the increased survival of infants born prematurely [18, 19]. However, the anticipated decrease in BPD after surfactant instillation has not been seen, as many preterms and especially extremely preterm infants are still at risk of developing BPD and long-term compromised lung function after surfactant therapy [10, 20]. Early studies by Goldsmith et al. [11] postulated that the immediate improvement in lung volume after exogenous surfactant instillation may be related to overdistension of alveoli rather than recruitment of functional alveoli based on markedly improved functional residual capacity (FRC) but with no change in lung compliance (C_L_) [11]. Similar changes in FRC were reported by Dinger et al. [21] in preterm infants after surfactant treatment for severe respiratory distress syndrome (RDS) [21]. Both these studies suggest that already at this early stage, there is a possible risk that surfactant might induce an overexpansion of the lung combined with atelectasis [11, 21]. In our present study, we wanted to investigate if computerized image analysis of chest radiographs (CXRs) from extremely preterm infants instilled with surfactant within 6 h could define similar changes in aeration in line with the lung mechanical changes previously described [11, 21].

Chest radiography is a widespread, inexpensive, and convenient method for assessing the lungs in the daily practice of neonatal intensive care. We found that conventional CXRs may contain additional and more detailed background information compared to what is currently provided by conventional radiological interpretation on the general appearance of the CXR. Our semiautomated digital analysis of CXRs could process a large number of images in an objective way without inter-observer variabilities. There are several studies that have used computerized image analysis of adult CXRs in combination with deep learning techniques to distinguish anatomical structures or disease types [22]. Xing et al. used a deep learning analysis of CXRs for early severity prediction of BPD by using size and angle measurements of anatomical structures [23]; however, we could not find any studies which interpret lung density measurements of extremely preterm infants with conventional CXRs by using digital image analysis techniques such as those employed in our study.

We found that the right lung has a lower MPI consistent with a higher index of aeration than in the left lung after surfactant treatment. MPI was higher in apical regions compared to basal in both the pre-surfactant (PSG) and surfactant-treated group (STG), suggesting that the conditions of cranio-caudal air distribution in the lungs are the same, regardless of surfactant treatment. A more aerated right lung could possibly be due to the lung anatomy with a more vertically directed right than left main bronchus [24–26], allowing airflow to reach the right side more easily. Studies have shown that there is a greater tendency to develop right-sided aspiration pneumonia which is also in line with the anatomical structure of the lungs [27]. Consequently, the anatomy of the lung could allow for more surfactant reaching the right lung and specifically the basal parts as observed in our study after surfactant replacement therapy.

Our findings suggest that the focal air distribution is more heterogeneous in the basal regions on the right side after surfactant replacement therapy. This is consistent with the interpretation that exogenous surfactant reaches the anatomically more available right basal side to a greater extent than the left. This is also consistent with the postulate that exogenous surfactant reaches the already patent alveoli more easily. A higher heterogeneity of aeration (FHPI) is also consistent with what Goldsmith et al. had previously suggested [11], that the focal heterogeneous air distribution reflects a combination of more pronounced over-expanded and collapsed alveoli because of uneven surfactant distribution. An uneven distribution of aeration with more pronounced over-expansion of patent alveoli might further compress the already collapsed adjacent alveoli. Physiologically, this would be consistent with Laplace’s law, which states that the pressure required to maintain the patency of an alveolus (Δ*P*) is directly proportional to the surface tension (*γ*) and inversely proportional to the alveolar radius (*r*) (Δ*P* = 2γ/r) [28]. Hence, smaller alveoli require a lower surface tension and would therefore need higher concentrations of surfactant compared to larger alveoli to remain open. We hypothesize that intratracheally administered surfactant might be more easily distributed to larger and more open alveoli than to smaller and collapsed ones.

The heterogeneity of aeration (FHPI) was higher in apical regions compared to basal regions in the pre-surfactant group (PSG). This is consistent with the notion that RDS lungs present with classical surfactant-deficient regions characterized by marked heterogeneous air distribution in the less aerated apical regions compared to the more aerated basal regions. However, the indication for surfactant treatment, with high oxygen need and/or clinical signs of respiratory distress, was still not met in the PSG infants at the time of the CXR, showing the discrepancy between clinical signs of surfactant deficiency and a more sophisticated radiological analysis.

On day 1, index of aeration (MPI) correlated to mean airway pressure (MAP) whereas it did not correlate to FiO_2_. This is noteworthy as FiO_2_ and work of breathing are the two main criteria recommended as clinical surfactant replacement therapy indications, according to the European consensus guidelines [7]. However, it is well established that MAP positively correlates with oxygen saturation levels in RDS, and given that there was heterogeneity in aeration even after surfactant therapy, higher MAP may be needed in lungs with less aeration (i.e., higher MPI) to open up collapsed alveoli. Thus, MAP could be more prognostic than FiO_2_ for indicating severity of RDS, and in our study positively correlated with lung aeration. This is also consistent with the relatively high Respiratory severity score seen in the surfactant treated patients in our cohort compared to other recently published cohorts [29].

We also found that overall MPI correlates with RDS severity, confirmed by the general image assessment of CXRs made by two pediatric radiologists. This indicates that the computerized image analysis of CXRs is comparable to clinical assessment. Furthermore, it provides more detailed information on air distribution in the different parts of the lungs, in addition to defining local heterogeneity of air distribution with FHPI, compared to classical radiological interpretation of RDS.

Overcoming heterogenous surfactant distribution has been a challenging aspect ever since infants started to be treated with surfactant. Changed position of the patient with separate instillations of the right and left lung with a slow instillation was proposed during the early synthetic surfactant studies [30], the latter with the intention that an initial slow recruitment of the lung might enable a more even distribution of surfactant. A fast instillation was established with natural surfactant, where the increased pressure during instillation was intended to overcome both differences in anatomy and compliance between the right and the left lung [31, 32]. Another suggested approach was to instill surfactant early while there still was a homogeneous liquid layer left, whereby the surfactant was hoped to be dispersed more evenly [33]. Inhalation of surfactant is yet another mode that has been proposed both as a non-invasive form of surfactant treatment and as a more evenly dispersed surfactant treatment [34]. Due to technical problems in executing inhalations of surfactant and the prerequisite to try to recruit more lung prior to inhalations, this mode has not yet reached a clinical application. The idea of aerating the lung before instilling surfactant is partly contradicted by our findings from CXRs taken prior to surfactant instillation, where a similar difference between the right and the left lung, and between the apical and the basal parts, was still observed. The earlier the instillation of surfactant is given in established RDS, the better the outcome might indicate that the lung might be more homogeneously aerated in the early phase of RDS, but this needs to be more thoroughly studied. The possible beneficial effects of not only avoiding invasive mechanical ventilation but also instilling surfactant during non-invasive ventilation (LISA and MIST) need also to be more thoroughly studied, as partially negative pressures extending the lung during inspiration might give a more even pressure distribution of the lung with possibly a more even surfactant distribution of the lung [35]. The timing of surfactant instillation both during invasive and non-invasive ventilation and different types of ventilatory modes prior to instillation are further fields to be explored.

Computerized image analysis of CXR may support new knowledge on lung physiology and surfactant replacement therapy, and could help to evaluate the different non-invasive applications of surfactant administration such as less-invasive surfactant administration and minimally invasive surfactant therapy compared to INtubation-SURfactant-Extubation in future studies. There are currently few studies on automated image analysis of CXR assessment in humans, and the existing ones have mainly studied surfactant distribution in animals. Cassidy et al. studied two different methods of surfactant replacement therapy in mice using microfocal CXR, and similar to our findings, they found that repeated surfactant doses tend to be distributed to the already patent alveoli rather than opening new ones [12].

### Strengths and weaknesses of the study

The strength of the study is the similar GAs and timing of CXRs in both the surfactant-treated and the pre-surfactant group. All infants were selected from the same center; hence, the infants were treated with the same protocol and CXR policy. An advantage with our method is the objectiveness of measurement by computerized image analysis. A potential disadvantage of the FHPI measurements is related to the size of the chosen region of interest (ROI), where a larger one would be expected to yield a higher heterogeneity of aeration (FHPI) value simply due to including a larger number of pixels, as exemplified by the basal region of segments 6 and 7. The FHPI results of the pre-surfactant group, however, seem to refute this notion since the heterogeneity of aeration was rising towards the apical regions where ROIs were generally smaller compared to the basal regions.

There were some weaknesses in our study related to the initial selection of infants, which was based on whether they had received surfactant or not, and CXRs were selected based on proximity to surfactant instillation in order to visualize surfactant distribution at an early stage, i.e., within 6 h, and also in view of the earlier lung mechanical studies after surfactant instillation [11, 21]. Infants in the PSG cannot therefore be defined as true controls since they likely included infants with less severe initial RDS that did not fulfill clinical criteria for early surfactant replacement therapy, and therefore had delayed surfactant instillation. However, given the known benefits of surfactant therapy, there is no expectation that there would be anyone in this modern cohort of extremely preterm patients with increased WOB and FiO2 requirement who would not be given surfactant. PSG did fulfill the criteria of having a CXR before 6 h of age similar to STG, thereby enabling some comparison of lung aeration at this early stage and similar postnatal age between the two groups. As the CXRs are mainly made to assure right placement of umbilical catheters according to the local clinical policy, no additional CXR was made in the PSG after instillation even though all received surfactant treatment.

We could not confirm any associations between MPI or FHPI and long-term outcomes characteristics in our study as too few patients were included to allow for confident correlations to be made; but this needs to be studied in larger cohorts in the future. We believe that digital image analysis tools as described in this study using routine CXR evaluations in preterm neonates, may have excellent potential for comparing surfactant administration methods such as less invasive surfactant administration, minimally invasive surfactant therapy, and INtubation-SURfactant-Extubation, and may even enable prediction of long-term outcomes in further follow up studies.

More elaborated investigations such as computerized tomography (CT) scans, magnetic resonance imaging (MRI), and electric impedance tomography (EIT) could probably provide more data, specifically on lung volumes. CT scans still need an automated image analysis to objectivize the information on heterogeneity of air distribution, but is presently not fully available bedside and add increasing radiation exposure to the patient. Promising bedside devices such as ultrashort echo time MRI are under way and include sophisticated automated image analysis, with the benefit of no radiation exposure [36]. EIT is starting to be applied on infants < 750 g, but more detailed assessment of heterogeneity needs further software development. Lung ultrasound is another promising bedside method, but would have to be validated as to measuring heterogeneity at the level of pixel intensity, which allowed analysis of both focal heterogeneity and mean air distribution in the present study on CXRs.

## Conclusion

Basal regions of the lungs were more aerated than apical ones, regardless of surfactant treatment, and the right lung was more aerated than the left after surfactant treatment. We hypothesize that surfactant probably reaches the right side more easily and the basal regions of the lung to a greater extent than the apical ones, leading to focal heterogeneous air distribution that might reflect a combination of both over-expanded and collapsed alveoli, observations that warrant continued future studies. Computerized image analysis of CXRs corresponded with conventional clinical radiological grading of CXRs. The optimal timepoint and the ideal administration method of surfactant in RDS need to be further investigated, wherein computerized image analysis of CXRs might provide a simple and powerful tool for this evaluation.

## Supplementary Information

Below is the link to the electronic supplementary material.

ESM 1 (PDF 762 KB)

## Data Availability

Collected clinical data and results from chest X-rays are available upon request. No AI was used for the study.
