# Supplementary material for: Computerized chest radiograph analysis of air distribution after surfactant treatment of respiratory distress syndrome in extremely preterm infants
Source: Eur J Pediatr. 2025 Oct 14;184(11):690. doi: 10.1007/s00431-025-06466-1 (PMC12521333; doi:10.1007/s00431-025-06466-1)
Supplement: Supplementary file 1 — (PDF 762 KB) [file 431_2025_6466_MOESM1_ESM.pdf]

Computerized chest radiograph analysis of air distribution after surfactant treatment of respiratory distress syndrome in extremely preterm infants

Julia Fall[1] MD, Richard Sindelar[1] MD PhD, Malin Helenius[2] MD PhD, Eva Penno[2] MD PhD, Leif D Nelin[1,3] MD, Laszlo Markasz[1*] MD PhD

[1]Department of Women's and Children's Health, Uppsala University, Uppsala, Sweden

[2]Department of Surgical Sciences; Radiology, Uppsala University, Uppsala, Sweden

[3]Nationwide Children's Hospital, Ohio State University College of Medicine, Columbus, Ohio, USA

***Corresponding author:** Laszlo Markasz

Supplement 1. Selection of infants and chest radiographs.

Surfactant Treated Group (n=52) with chest radiographs <6 hours after surfactant treatment. Pre-Surfactant Group (n=8) with the earliest chest radiograph, taken prior to surfactant administration. ELGANs=Extremely Low Gestational Age Newborns; NICU=Neonatal Intensive Care Unit

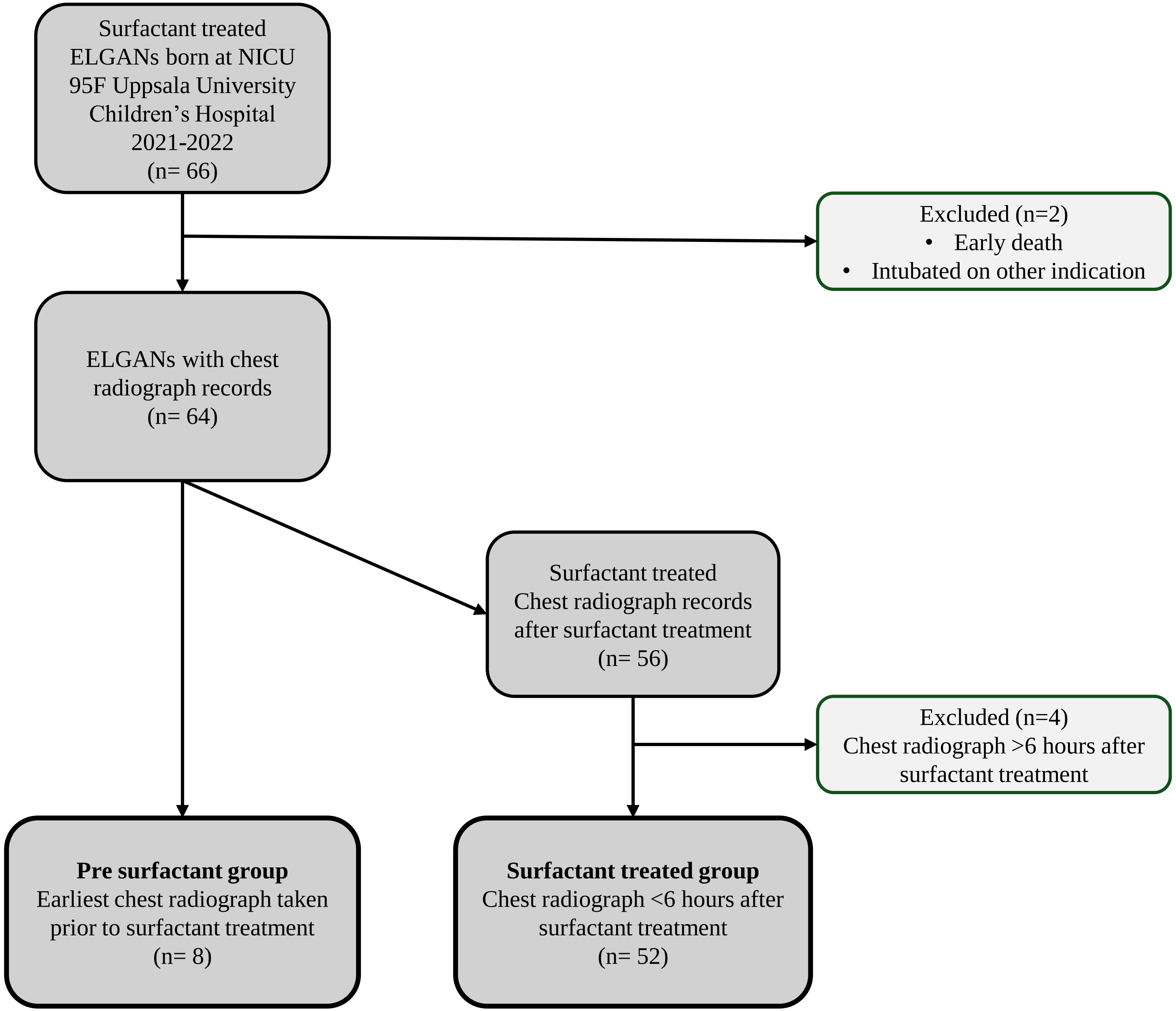

Supplement 2A-B. Intensity and size normalization, and ROI selection (Patient nr42).

Arrow: Selection of the reference point with the maximum intensity region of the liver. Each image was normalized for the pixel intensities according to the respective reference point. A scale bar of 10 mm helped to adjust the image size for each image to make the area measurements between infants comparable. 50 pixels = 10 mm. Twelve Region of Interests (ROIs), according to the intercostal space 2 to 7 on the left and right lung respectively, were selected manually for each infant by avoiding other structures than lung tissue (**Supplement 2A**). The pixel intensity levels simulated with colors in **Supplement 2B**.

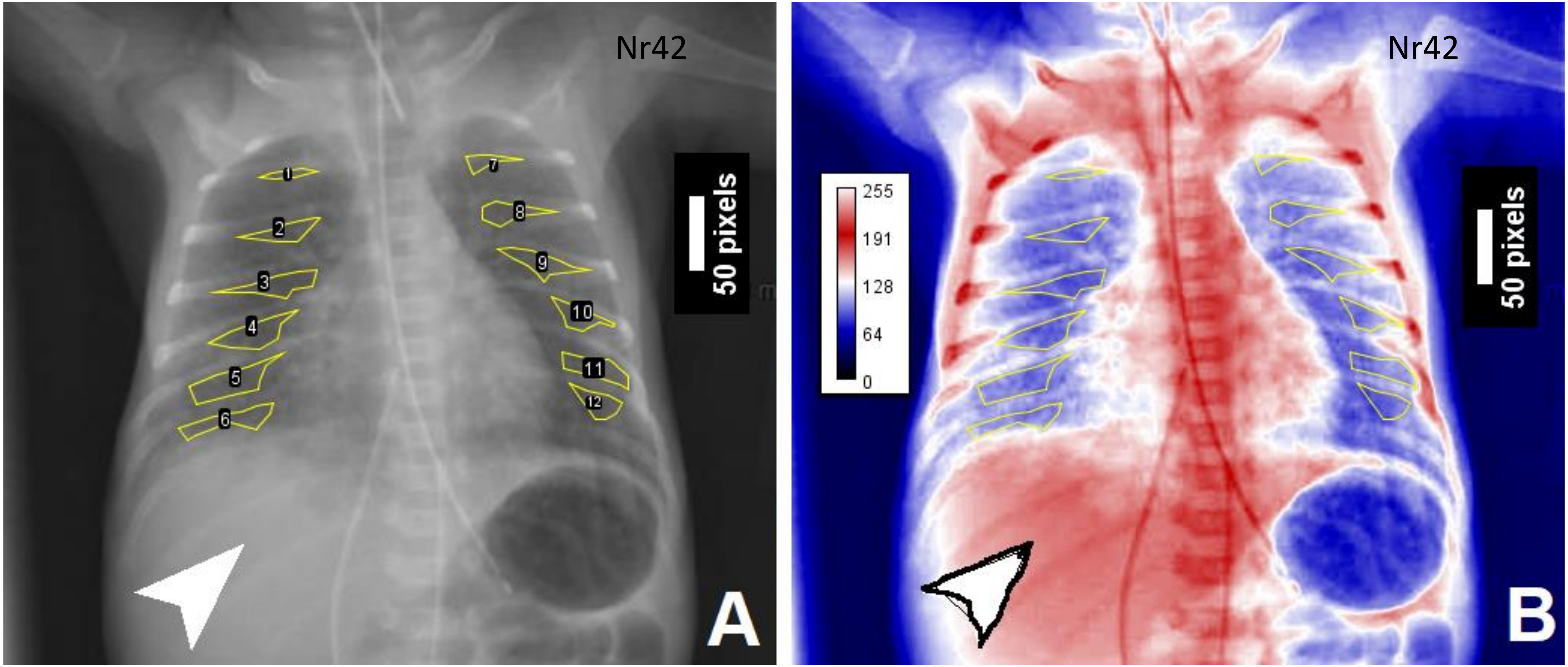

Supplement 3A-E. Lung density measurement.

A-C: Illustrate a representative infant with low mean pixel intensity (MPI), low lung density in the right lung (Patient nr12), corresponding to high air content.

D-F: Illustrate a right lung field with high MPI, high lung density (Patient nr14), corresponding to hypoinflation (low air content).

Right MEAN: Calculated from the MPI of the ROIs of the right lung.

B and E: Pixel intensities are simulated with colors. Blue corresponds to low and white/red-white to high density (low air content).

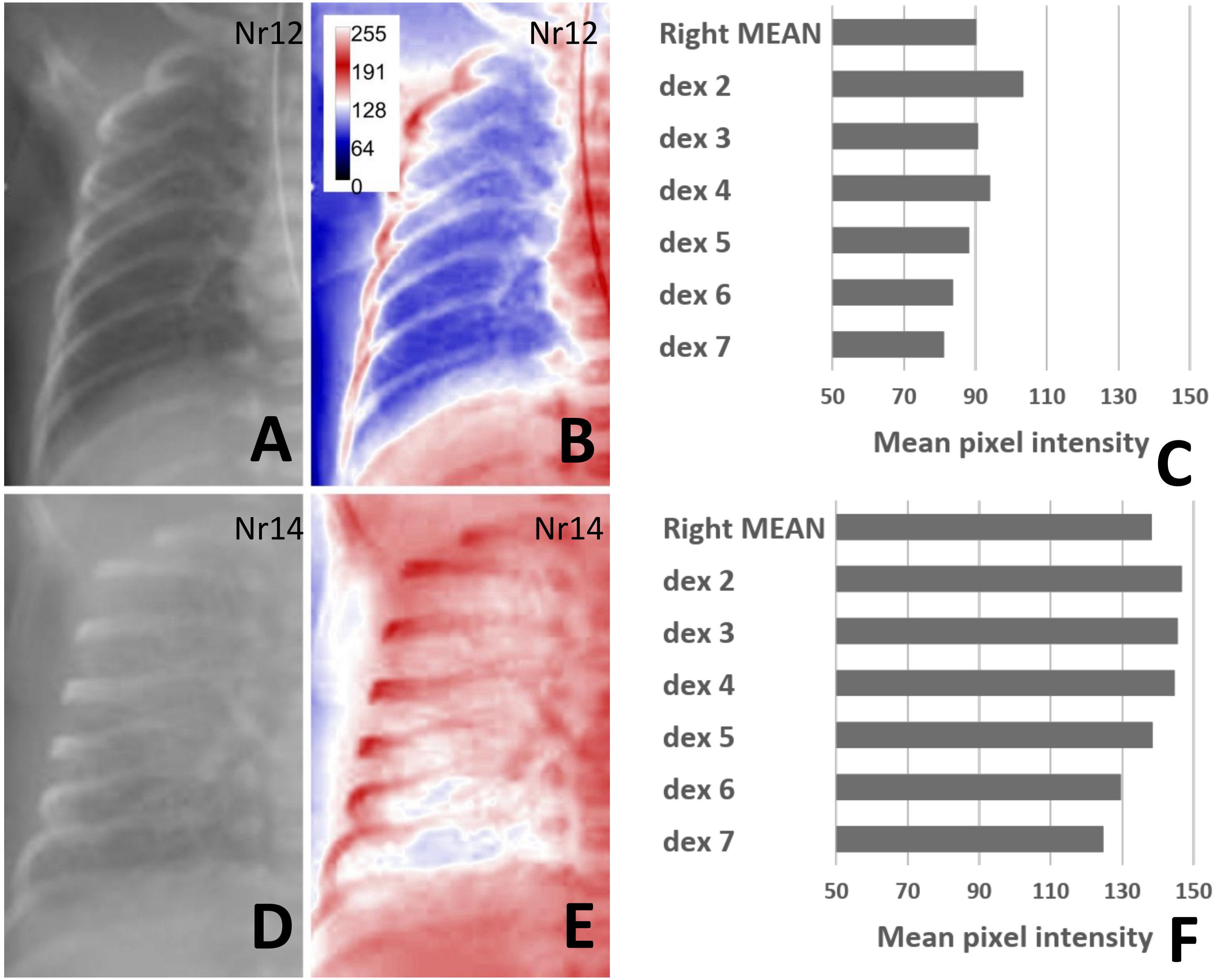

Supplement 4A-E. The Focal Heterogeneity in Pixel Intensities (FHPI).

The focal pattern of heterogeneity in the corresponding lung segment. Higher FHPI reflects a higher focal heterogeneity of air distribution in the individual lung segment.

A: The right lung of Patient Nr10 with the selected ROI.

B: The 7th right intercostal segment with low FHPI. Intensity differences simulated with colors (the higher the difference in color, the higher the difference in pixel intensity).

C: The right lung of Patient Nr52.

D: The 7th right intercostal segment with high FHPI (the higher the difference in color, the higher the difference in pixel intensity).

E (graph): FHPI of the 7th right intercostal segment in Patient Nr10 and Nr52.

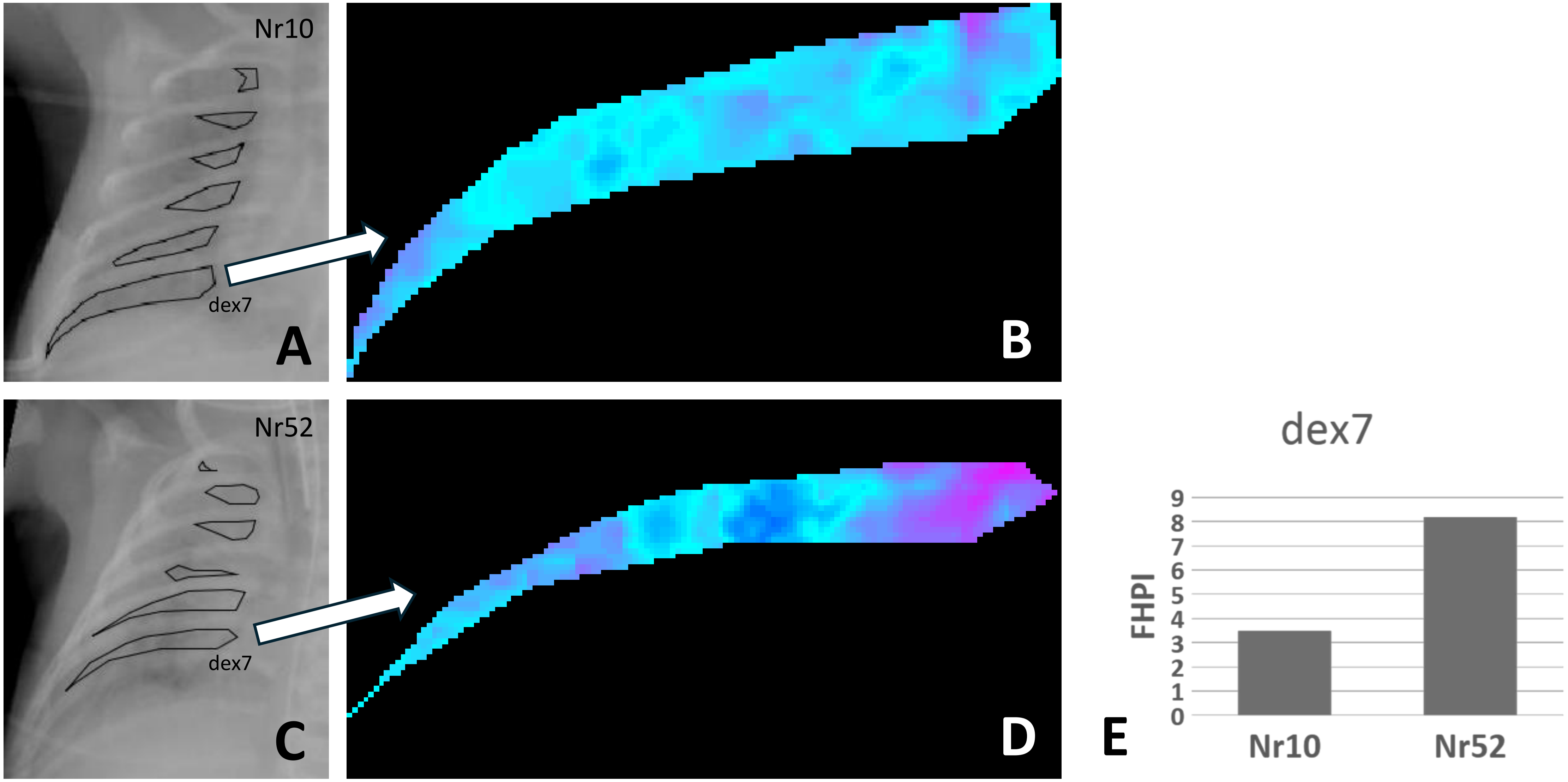

Supplement 5A-G. Heterogeneity of air distribution in entire lung fields (STDV-MPI).

High standard deviation of mean pixel intensity (STDV-MPI) illustrates a high heterogeneity (uneven air distribution) of entire lung fields (Patient Nr39 in G). Blue corresponds to low and white/red-white to high density (low air content).

A-C: Right lung field with low heterogeneity (low STDV-MPI) in Patient Nr29, reflecting an even air distribution.

D-F: Right lung field with high heterogeneity (high STDV-MPI) in Patient Nr39, reflecting an uneven air distribution with higher lung density in the apical region of the lung.

G: STDV-MPI of the right lung in Patients Nr29 and Nr39.

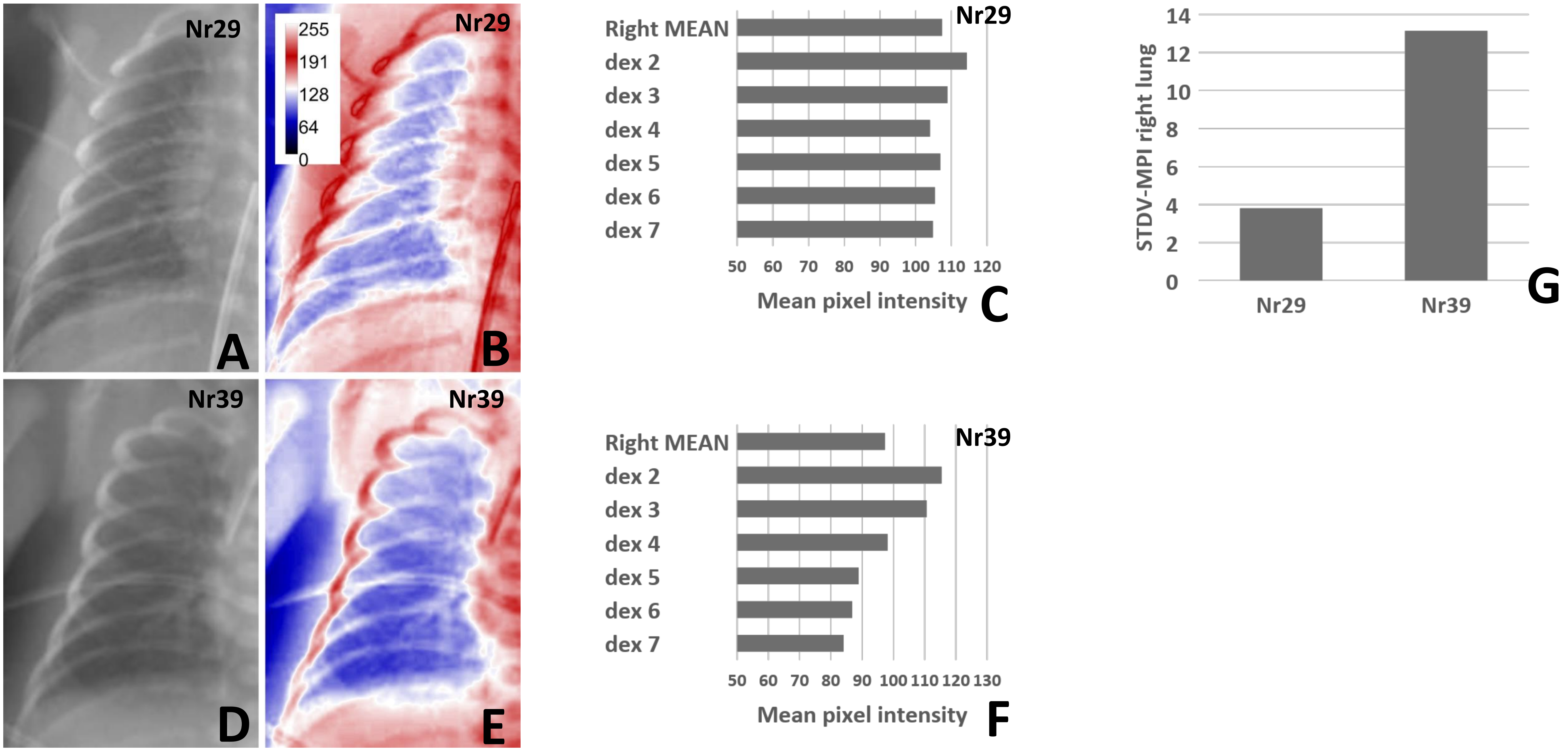

Supplement 6A-I. Standard deviation of FHPI (STDV-FHPI).

Standard deviation of focal of heterogeneity (STDV-FHPI) among the lung segments. Higher STDV-FHPI corresponds to higher differences in local air heterogeneity among the lung segments.

A-D: The right lung of Patient Nr10 with low FHPI and homogenous distribution among the lung segments, resulting in low STDV-FHPI.

E-H: The right lung of Patient Nr52 with both high and low FHPI which result in high STDV-FHPI and heterogeneous appearance of the lung structure among lung segments.

A, E: Original images with ROI. D, H: FHPI levels of the lung segments in Patient Nr10 (D) and Nr52 (H). Right MEAN: Calculated from the STDV-FHPI of the ROIs of the right lung.

I: The mean of STDV-FHPI levels of Patients Nr10 and 52.

B, F: Pixel intensity levels are simulated with colors similarly as in Supplement 2, 3, 5.

C, G: Intensity differences in the selected ROI of intercostal segments simulated with colors as in Supplement 4 (more different colors correspond to higher differences in intensities in the same ROI).

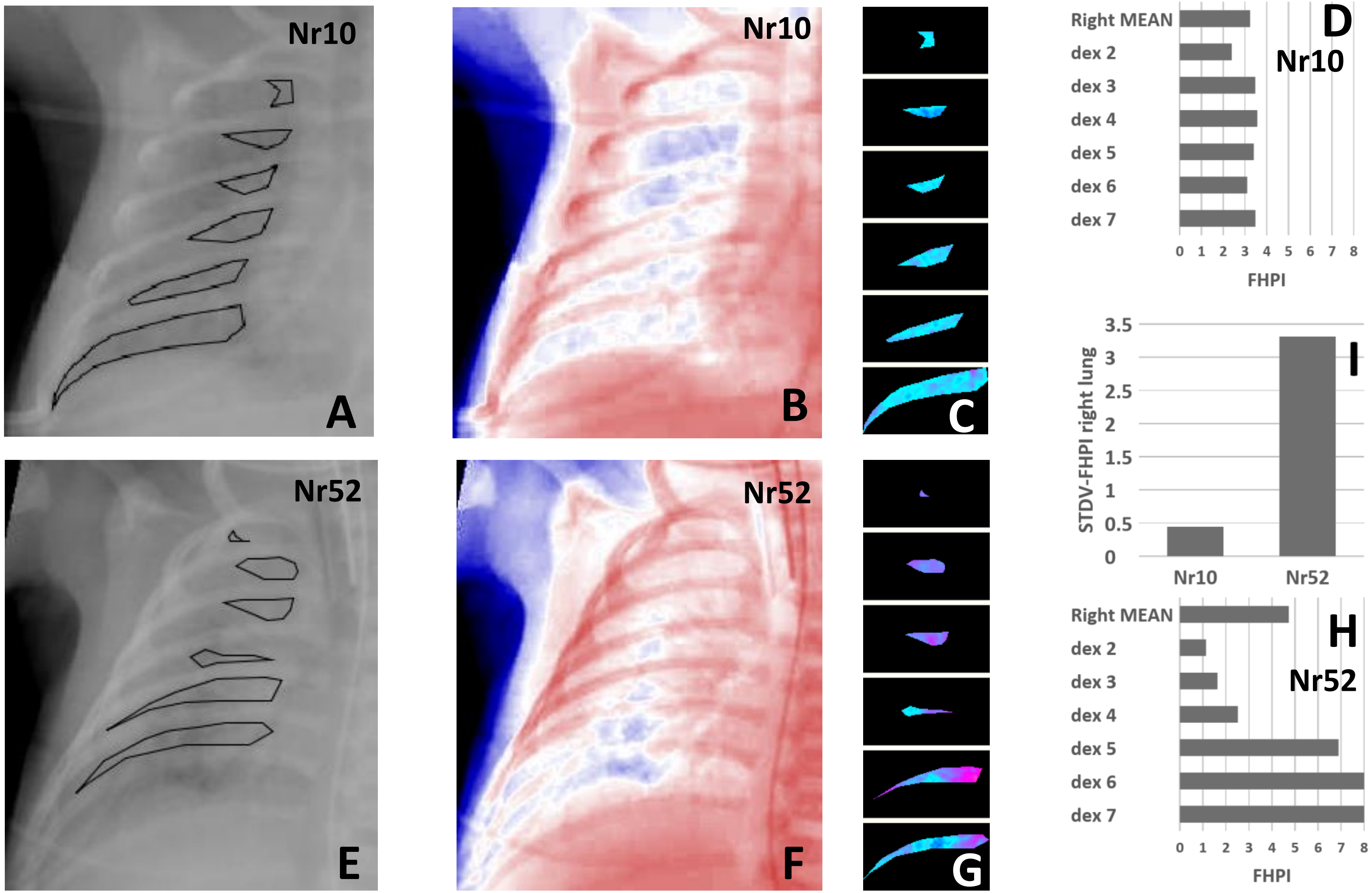

Supplement 7. Number of infants in STG on MV and NIV mode respectively, number of infants in STG on supplementary oxygen supple and discharged.

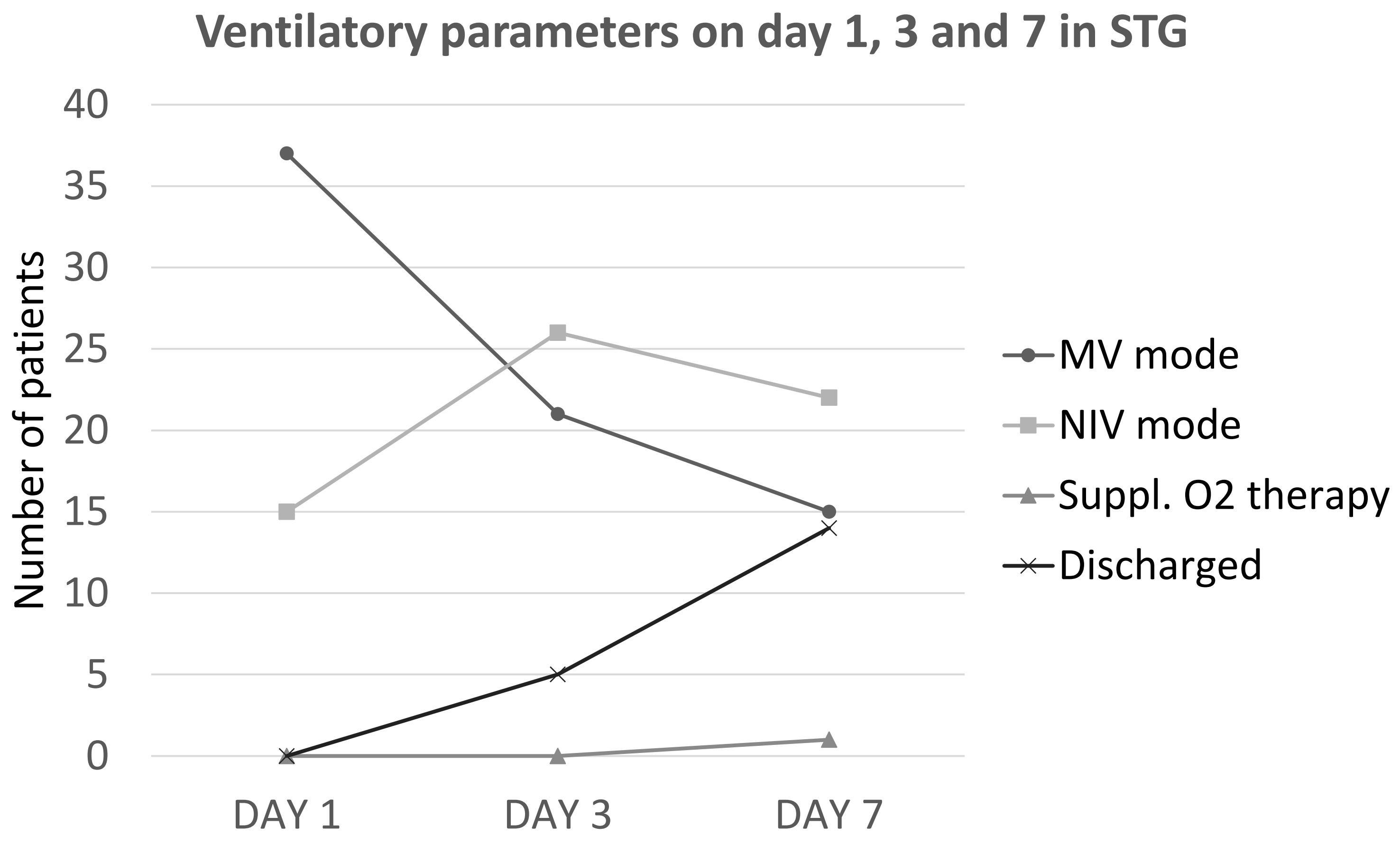

Supplement 8. Ventilatory parameters on day 1, 3 and 7 in the Surfactant Treated Group (n=52).

MV mode signifies ventilation via an endotracheal tube and includes HFOV/CPAP, Synchronized Intermittent Mandatory Ventilation (S-IMV) and Assist Control (A/C), with or without Pressure Support (PS). NIV mode signifies non-invasive ventilation and includes CPAP, nasal ventilation, or HFNC. *Supplementary O_2 therapy* includes infants not requiring any ventilatory support, only O_2 supplement via a low-flow nasal cannula. *Discharged* includes both deceased and infants moved to another hospital. Tidal volume (*VT*), positive inspiratory pressure (*PIP*), respiratory rate (*RR*), inspiratory and expiratory time (T_{insp}, T_{exp}) could only be extracted from infants on MV mode. Differences between day 1 and 3, 3 and 7, and 7 and 1 were estimated to be significant according to repeated measures ANOVA for parametric values or Friedman test for non-parametric values followed by the Bonferroni (p-bonf) or the Conover´s (p-con) post hoc tests respectively. MV=mechanical ventilation; NIV=non-invasive ventilation; MAP=mean airway pressure; FiO2=fraction of inspired oxygen; PIP=positive inspiratory pressure; PEEP=positive end-expiratory pressure; SD=standard deviation.

	**Day 1**	p-con	**Day 3**	p-con	**Day 7**	p-con
	n (%)		n (%)		n (%)	
**MV mode**	37 (71)	**<0.001**	21 (40)	0.308	15 (29)	**<0.001**
**NIV mode**	15 (29)	**0.014**	26 (50)	0.884	22 (42)	0.204
**Suppl. O_2 therapy**	0 (0)	1.000	0 (0)	0.670	1 (2)	0.670
**Discharged**	0 (0)	0.215	5 (10)	**0.004**	14 (27)	**<0.001**
	Mean ± SD	p-bonf	Mean ± SD	p-bonf	Mean ± SD	p-bonf
**MAP, cmH_2O**	6.98 ± 2.2	0.899	6.66 ± 2.3	1.000	6.82 ± 2.4	1.000
**FiO_2, %**	33 ± 16	1.000	32 ± 14	**0.029**	37 ± 21	0.251
**Respiratory severity score**	23.3 ± 16.8	1.000	21.1 ± 20.5	**0.037**	29.1 ± 27.2	0.534
**VT, ml/kg**	6.1 ±2 .7	**<0.001**	5.5 ± 2.2	1.000	6.3 ± 3.4	**<0.001**
**PIP, cmH_2O**	19.2 ± 4.0	0.777	21.2 ± 11.3	1.000	20.5 ± 5.0	**0.048**
**PEEP, cmH_2O**	4.9 ± 0.9	1.000	5.1 ± 1.2	1.000	5.3 ± 1.6	0.526
**RR, per minute**	64 ± 26	1.000	61 ± 7	**0.037**	60 ± 7	0.534
**T_{insp}, seconds**	0.30 ± 0.01		0.30 ± 0.00	0.669	0.30 ± 0.01	0.669
**T_{exp}, seconds**	0.67 ± 0.01	0.535	0.66 ± 0.02	1.000	0.66 ± 0.03	0.372

Supplement 9. Total MPI (left and right) correlation to MAP Day 1.
Only infants from the surfactant treated group were included (n=52). There was a significant positive linear correlation between total MPI and MAP on Day 1 ($r = 0.479$; $p<0.001$).

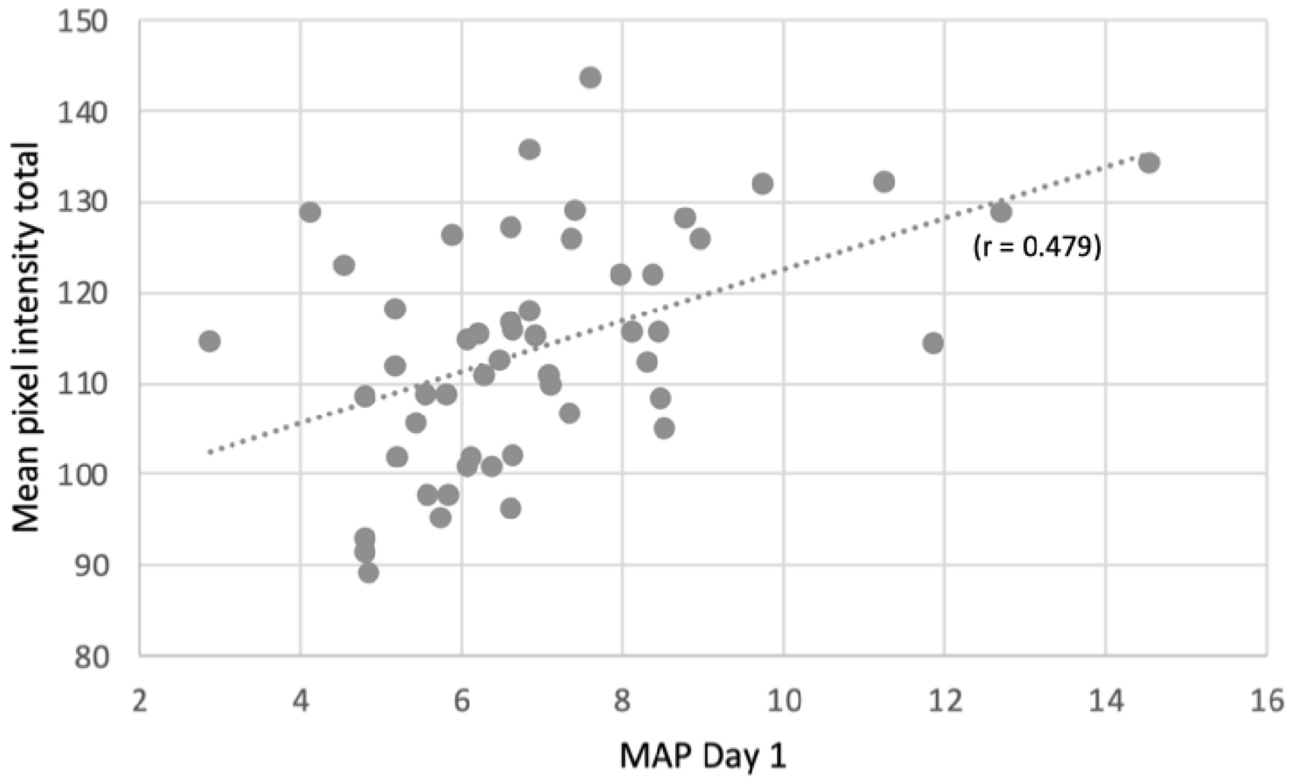

Supplement 10. Long-term outcomes in the Surfactant Treated Group (n=52)
Percentages calculated on the whole group (n=52).
IVH=Intraventricular hemorrhage; PDA=Persistent ductus arteriosus;
ROP=Retinopathy of prematurity; NEC=Necrotizing enterocolitis;
BPD= Bronchopulmonary dysplasia; sBPD=Severe bronchopulmonary dysplasia.

	**Mean ± SD**	
**Total ventilation duration in intubated infants, days**	20 ± 33	
**CPAP duration, days**	28 ±22	
**MV duration, days**	17±31	
**HFOV duration, days**	5 ±7	
**HFNC duration, days**	26 ±14	
**Length of stay, days**	75 ±51	
	**n**	**%**
**IVH grade 3-4**	5	10
**PDA, surgically treated**	2	4
**ROP grade 3-5**	4	8
**ROP, treated**	2	4
**NEC**	2	4
**NEC with perforation**	1	2
**NEC, surgically treated**	2	4
**BPD**	20	38
**sBPD**	12	23
**Deceased**	17	33
